# Eating Behavior, Body Appreciation, and Sleep Quality in Italian Blind Baseball Athletes: A Cross-Sectional Exploratory Survey

**DOI:** 10.3390/healthcare14203446

**Published:** 2026-10-15

**Authors:** Giuditta Carretti, Sofia Lotti, Monica Dinu, Francesco Sofi, Mirko Manetti, Barbara Colombini, Mirca Marini

**Affiliations:** Department of Experimental and Clinical Medicine, University of Florence, 50134 Florence, Italy; giuditta.carretti@unifi.it (G.C.); sofia.lotti@unifi.it (S.L.); monica.dinu@unifi.it (M.D.); francesco.sofi@unifi.it (F.S.); mirko.manetti@unifi.it (M.M.); barbara.colombini@unifi.it (B.C.)

**Keywords:** visually impaired athletes, blind baseball, adapted physical activity, lifestyle habits, eating behavior, sleep quality, body appreciation, health promotion

## Abstract

**Background/Objectives:** Visual impairment is associated with poor sleep quality, psychological distress, and unhealthy lifestyle behaviors. Adapted sports, such as blind baseball (BXC), may help promote healthy lifestyles and overall well-being. However, evidence regarding eating behavior, sleep quality, body appreciation, and lifestyle habits among regular BXC players remains scarce. This cross-sectional study provides an exploratory multidimensional characterization of these domains in competitive Italian BXC athletes according to sport participation and visual impairment characteristics, comparing findings descriptively with normative data from the Italian general population. **Methods**: Fifty-three competitive Italian BXC players (mean age 39.3 ± 13.1 years; representing a response rate of 49.1% of the national league) completed an anonymous online survey assessing eating behavior (Adult Eating Behavior Questionnaire, AEBQ), body appreciation (Body Appreciation Scale-2, BAS-2), sleep quality (Brief Pittsburgh Sleep Quality Index, B-PSQI), and lifestyle habits. Group differences were assessed using chi-square tests for categorical variables and Mann–Whitney *U* tests for continuous variables, reporting medians, interquartile ranges, effect sizes (rank-biserial r), and 95% confidence intervals. **Results:** BXC athletes demonstrated BMI, sleep quality, eating behavior, and body appreciation profiles comparable to those of the Italian general population. BAS-2 scores were high (3.7–3.8), while B-PSQI scores (4.0–5.6) and the proportion of poor sleepers (~40%) remained within normative ranges despite visual impairment. Eating behavior showed no significant dysregulation. However, in exploratory subgroup analyses, multisport athletes displayed lower Satiety Responsiveness than BXC-only athletes (2.30 ± 0.79 vs. 2.78 ± 0.71; *p* = 0.02), poorer sleep quality (B-PSQI: 6.05 ± 3.12 vs. 4.70 ± 3.73; *p* = 0.05), and a higher prevalence of poor sleep (55.0% vs. 30.3%). Athletes with acquired visual impairment showed higher Emotional Overeating and Emotional Undereating scores than those with congenital impairment, although these differences did not reach statistical significance. **Conclusions:** BXC athletes displayed an overall favorable lifestyle profile. Though the cross-sectional design prevents causal inferences, these exploratory findings indicate that participation in BXC is compatible with healthy lifestyle habits and positive body appreciation, hence supporting adapted team sports as an inclusive framework for multidimensional health promotion. Future studies using objective physiological assessments and longitudinal designs are needed to clarify dose–response relationships and long-term health outcomes.

## 1. Introduction

Visual impairment represents a major global public health challenge, with blindness affecting more than 43 million people and distance or near vision impairment affecting more than 2.2 billion individuals worldwide, drastically impacting psychophysical health, autonomy, social participation, and quality of life [1,2]. Beyond sensory and functional limitations related to disability, and regardless of age, blind and visually impaired individuals frequently experience disproportionately higher rates of physical inactivity, sedentary behavior, cardiometabolic disorders, psychological distress, and social isolation compared with the general population [3,4,5]. Furthermore, environmental and transportation barriers—along with limited access to sports facilities and tailored exercise programs—contribute to health inequalities by limiting opportunities to adopt a healthy lifestyle [6,7,8]. Consequently, promoting healthy habits through adapted physical activity/sport interventions has progressively become a priority within sensory disability research and global public health policies [7,9,10]. Physical activity can improve sleep quality, healthier eating behaviors support recovery and cognitive function, and adequate sleep contributes to emotional regulation, resilience, and overall self-regulation [11,12,13]. Conversely, unhealthy dietary habits, poor sleep, and sedentary lifestyles reinforce one another thus contributing to increase chronic disease risk and psychological distress [14,15]. This integrated perspective is particularly relevant in populations with visual impairment, in whom lifestyle interventions may simultaneously address multiple determinants of health.

The literature has increasingly recognized regular physical activity as one of the most effective non-pharmacological interventions to prevent chronic diseases and improve physical, cognitive, and psychological functioning [16,17,18]. In people with visual impairments, exercise and adapted sports have been associated with improved physical fitness, functional independence, self-efficacy, balance, and health-related quality of life [19,20,21,22]. Nevertheless, physical activity levels remain below international recommendations because of environmental and accessibility barriers [4,23,24,25], contributing to poorer health-related quality of life and increased cardiometabolic risk [26,27]. Inclusive sport programs are therefore important for overcoming such barriers and supporting long-term adherence to healthy behaviors [7,8]. Beyond physiological adaptations, sport participation promotes social integration, resilience, self-esteem, life satisfaction, and psychological well-being while reducing social isolation [28,29]. Systematic reviews have highlighted that parasport participation is associated with higher self-efficacy, social connection, and overall well-being [29,30,31]. In particular, team sports can potentially strengthen group identity, social support, and inclusion [32,33]. Such mechanisms are especially important for visually impaired individuals, who often experience reduced opportunities for spontaneous interaction, primarily due to communication barriers and diminished access to visual social cues [34].

Among adapted team sports, blind baseball (BXC) represents a unique Italian model of inclusive physical activity—purposefully designed for visually impaired athletes—that combines auditory cues, proprioceptive orientation, spatial cognition, and cooperative tactical strategies, enabling athletes with different levels of visual impairment to compete together under standardized rules [35]. Growing evidence indicates that BXC produces benefits extending well beyond sporting performance. Previous studies have demonstrated higher psychological well-being and quality of life among Italian players compared with visually impaired sedentary controls [36], as well as kinesiological improvements in balance, proprioception, reactive agility, and upper-body functionality [37,38,39]. Regular participation also promotes empowerment, social inclusion, and gender equality [40]. Together with the intermittent high-intensity activity, sensorimotor integration, decision-making, and interpersonal interaction required by the discipline [41], these characteristics make BXC a relevant setting for describing lifestyle and psychosocial dimensions in visually impaired athletes.

Specifically addressing athlete health, current frameworks recognize that health outcomes emerge from an integrated 24 h movement continuum—where exercise, dietary behaviors, body appreciation, and sleep interact to support physical and psychological functioning [42,43]. Appropriate dietary habits support energy availability, recovery, body composition, metabolic health, and athletic performance [44,45,46,47]. However, para-athletes may face limited access to individualized dietary counselling, disability-related demands, and practical barriers to food preparation and dietary autonomy [48]. These challenges may be particularly relevant for visually impaired individuals, who can experience difficulties with food shopping, selection, label reading, meal preparation, and healthy food choices [49,50]. Despite these concerns, evidence on these key aspects among competitive blind athletes remains extremely limited.

Body appreciation, defined as accepting, respecting, and holding a positive attitude toward one’s body despite imperfections, constitutes another fundamental yet under-investigated component of a healthy lifestyle in adapted sport. Positive body image has been associated with greater psychological resilience, healthier eating behaviors, enhanced self-esteem, and improved sport confidence, whereas body dissatisfaction is linked to disordered eating, impaired mental health, reduced well-being, and poorer athletic outcomes [51,52,53,54]. While body image in visually impaired individuals is constructed through multisensory, tactile, and functional experiences rather than visual cues alone [40,55], evidence regarding body appreciation in competitive blind athletes remains limited [48].

Sleep quality represents a further pillar of athlete health alongside exercise and nutrition, influencing physical recovery, immune function, cognitive performance, emotional regulation, and injury prevention [47,56,57]. Growing evidence indicates that physically active individuals generally report better sleep quality than sedentary peers [11,13,58] and that adequate sleep contributes not only to athletic performance but also to psychological well-being and resilience [59,60]. Conversely, sleep disturbances impair recovery, increase injury risk, and negatively influence mood and decision-making [60]. These issues may be particularly relevant for individuals with blindness, because impaired light perception can disrupt circadian entrainment and the sleep–wake cycle [61].

Overall, adapted sports may provide important opportunities for physical activity and social inclusion among people affected by visual impairment. However, whether competitive BXC athletes also show favorable profiles of eating behavior, body appreciation, and sleep quality remains insufficiently documented. No previous study has simultaneously characterized these three domains in competitive visually impaired athletes, despite their relevance to physical performance, psychological well-being, and health-related quality of life.

To address this knowledge gap, the present study aimed to provide an exploratory, multidimensional characterization of eating behavior, body appreciation, and sleep quality among Italian competitive BXC players. In accordance with the 24 h movement framework, these three domains were evaluated as independent complementary indicators of a health-related lifestyle profile. Furthermore, to explore potential behavioral and psychological variations, exploratory subgroup analyses were conducted according to specific features. Specifically, we evaluated sport involvement (BXC-only vs. multisport), hypothesizing potential differences in recovery and appetite regulation due to training demands, as well as impairment onset (congenital vs. acquired), hypothesizing potential differences in emotional adaptation to disability. By exploring these complementary dimensions within one of the largest organized cohorts of visually impaired athletes, this study seeks to characterize an under-investigated population and provide foundational data for future longitudinal research aimed at designing tailored on-field interventions to foster healthy lifestyles and psychophysical well-being.

## 2. Materials and Methods

### 2.1. Study Design and Participants

A cross-sectional web-based investigation was conducted between 30 January and 15 March 2026 during the pre-season phase of the Italian BXC championship. The study target population comprised all 108 competitive players regularly registered with one of the Italian teams officially affiliated with the Italian Blind Baseball Association and League (AIBXC/LIBCI). A total of 53 competitive players voluntarily participated and completed the survey, yielding a response rate of 49.1%. The study cohort demonstrated demographic representativeness of the national league regarding age distribution, sex ratio (~77% male), and visual impairment severity (~85% legally blind). Data were collected fully anonymously using Google Forms (Google LLC, Mountain View, CA, USA), without requesting personal identifiers, contact details, or IP addresses. Nevertheless, since the research targeted a specific athletic population, the study design, survey items, and accessibility features were formally reviewed, endorsed, and approved by the internal review board of the AIBXC (approval number: 2026/29.01; approval date: 29 January 2026), the national governing body for blind baseball in Italy responsible for protecting athlete welfare and data privacy. The survey link was distributed through the official AIBXC communication channels and participants accessed it using assistive technologies, including screen readers (NVDA, NV Access, Camp Mountain, QLD, Australia; JAWS, Freedom Scientific, St. Petersburg, FL, USA; VoiceOver, Apple Inc., Cupertino, CA, USA) and digital accessibility tools previously described in our studies [36,62]. Visual impairment classification was self-reported by participating athletes based on Italian Law 138/2001 [63] (categorized for study purposes as legally blind or visually impaired), given that official BXC regulations, both nationally and internationally, do not adopt standard IBSA sports classification. Electronic informed consent was obtained from all respondents prior to survey initiation. The study was conducted in accordance with the ethical principles of the Declaration of Helsinki [64].

### 2.2. Data Collection

The questionnaire was structured into several distinct sections. The first section collected sociodemographic characteristics, including sex, age, marital status, and educational level, as well as anthropometric data (self-reported weight and height) and information regarding the type and severity of visual impairment. Body mass index (BMI, kg/m^2^) was calculated from the weight and height data. The questionnaire also included items assessing the participants’ sports background, including years of BXC practice and average weekly training hours. Dietary habits were investigated through questions regarding adherence to a specific dietary regimen during the previous year and the underlying reasons (if applicable), the frequency of eating meals outside the home, the frequency of cooking meals at home, and the habitual consumption of alcoholic beverages. The second part of the questionnaire comprised a series of previously validated instruments assessing nutrition-related knowledge and behaviors, body perception, and sleep quality. A detailed description of these measures is provided in the following sections.

#### 2.2.1. Eating Behavior

Individual eating behavior traits were assessed using the Adult Eating Behavior Questionnaire (AEBQ), a validated 35-item instrument comprising eight subscales grouped into two broader domains [65]: Food Approach and Food Avoidance. The Food Approach domain includes four subscales: Food Responsiveness (4 items), which reflects responsiveness to external food cues; Hunger (5 items), assessing the tendency to experience feelings of hunger; Emotional Overeating (5 items), evaluating increased food intake in response to negative emotions; and Enjoyment of Food (3 items), measuring general interest in and pleasure derived from eating. The Food Avoidance domain consists of four subscales: Satiety Responsiveness (4 items), which assesses sensitivity to feelings of fullness; Food Fussiness (5 items), reflecting reluctance to try or consume unfamiliar foods; Emotional Undereating (5 items), evaluating reduced food intake during emotional distress; and Slowness in Eating (4 items), assessing the tendency to eat at a slower pace. Responses were recorded on a 5-point Likert scale ranging from 1 (“strongly disagree”) to 5 (“strongly agree”). Mean scores for each subscale were calculated according to the scoring procedure described in the original validation study [65]. Internal consistency in the present sample was assessed using Cronbach’s alpha, with coefficients of 0.77 for Enjoyment of Food, 0.94 for Emotional Overeating, 0.96 for Emotional Undereating, 0.87 for Food Fussiness, 0.71 for Food Responsiveness, 0.92 for Slowness in Eating, 0.65 for Hunger, and 0.79 for Satiety Responsiveness. Published reference values for healthy Italian adults were used as an exploratory descriptive benchmark. In detail, mean AEBQ scores reported in such a target were 2.89 ± 0.82 for Food Responsiveness, 2.67 ± 1.16 for Emotional Overeating, 4.10 ± 0.71 for Enjoyment of Food, 2.88 ± 1.13 for Emotional Undereating, 2.11 ± 0.91 for Food Fussiness, 2.49 ± 1.01 for Slowness in Eating, and 2.38 ± 0.78 for Satiety Responsiveness [66].

#### 2.2.2. Body Appreciation

Body appreciation was investigated using the Body Appreciation Scale-2 (BAS-2), a validated self-report instrument developed to measure positive body image [67,68]. The BAS-2 consists of 10 items evaluating body acceptance, respect, and appreciation, as well as the ability to maintain a positive attitude toward one’s body despite its perceived imperfections. Participants rated each statement on a 5-point Likert scale ranging from 1 (“never”) to 5 (“always”). Item scores were averaged to obtain an overall body appreciation score, with higher scores indicating greater body appreciation. The BAS-2 has demonstrated robust psychometric properties across different populations and has been widely used in body image research. The Italian version demonstrated a unidimensional factor structure, with adequate internal consistency and evidence of construct validity. Internal consistency of the BAS-2 in the present sample was high (Cronbach’s alpha = 0.92). Published normative data for Italian adults served as a descriptive benchmark. Specifically, such reference values were 3.74 ± 0.73 for men and 3.93 ± 0.71 for women [69].

#### 2.2.3. Sleep Quality

The Brief version of the Pittsburgh Sleep Quality Index (B-PSQI) was used to evaluate overall sleep quality over the previous month [70]. It comprises six items assessing five dimensions of sleep: subjective sleep quality, sleep latency (in minutes), sleep duration (in hours), sleep efficiency, and the frequency of nocturnal awakenings. Each dimension is scored on a 4-point scale ranging from 0 to 3, with higher scores indicating greater sleep difficulties. The sum of the five subscale scores yields a global score ranging from 0 to 15, with higher scores reflecting poorer sleep quality. The B-PSQI was developed from the original Pittsburgh Sleep Quality Index and has shown satisfactory psychometric properties, including adequate internal consistency and validity, while providing a more time-efficient assessment of sleep quality [70]. Internal consistency of the B-PSQI in the present sample was acceptable (Cronbach’s alpha = 0.76). Published Italian population normative data (mean 4.10 ± 2.94 with a cut-off score of >5 indicating poor sleep quality) [71] were used for descriptive context.

### 2.3. Statistical Analysis

Data analysis was performed using IBM SPSS Statistics for macOS, version 28.0 (IBM Corp., Armonk, NY, USA). Continuous variables were presented as mean ± standard deviations (SD), whereas categorical variables were reported as frequencies and percentages. Normality of continuous variables was assessed using the Shapiro–Wilk test. For continuous variables included in non-parametric comparisons, median and interquartile range (IQR) were also reported alongside mean ± SD. Statistical significance was set at *p* < 0.05. Two subgroup analyses were conducted to examine potential differences in key study outcomes according to participant characteristics that might influence eating behavior, sleep quality, and body image. Specifically, analyses were stratified by sport involvement (BXC only vs. multisport) and by the onset of visual impairment (congenital vs. acquired). The two subgroup classifications showed substantial overlap: among BXC-only athletes, 21 had congenital and 12 acquired visual impairment, while among multisport athletes, 12 had congenital and 8 acquired impairment (Fisher’s exact *p* = 1.00). Differences between groups were assessed using the chi-square (χ^2^) test for categorical variables and the non-parametric Mann–Whitney U test for continuous variables. Given the sample size and the overlap between subgroup classifications, subgroup analyses were considered exploratory and descriptive rather than confirmatory, and individual *p*-values were interpreted accordingly, without formal adjustment for multiple testing. For the main continuous subgroup comparisons reported in the Results, rank-biserial correlation (r_rb) with 95% confidence intervals (CI) was used to describe the magnitude and precision of the observed differences.

## 3. Results

A total of 53 competitive Italian BXC players participated in the study, anonymously completing the structured online survey. The sociodemographic and anthropometric characteristics of the study sample are summarized in Table 1.

Briefly, the mean age of the sample was 39.3 ± 13.1 years, and players were predominantly male (77.4%). No significant differences in sociodemographic characteristics were observed between congenital and acquired visual impairment groups. The majority of participants were legally blind and had a congenital visual impairment. Regarding their anthropometric profile, the majority of athletes were classified as normal weight, whereas approximately one-third were overweight, and only a small proportion were classified as obese (Table 1).

As shown in Table 2, all participants regularly practiced competitive BXC, with mean weekly training duration of 3.49 ± 1.31 h.

In particular, approximately two-thirds (62.3%) participated exclusively in BXC, whereas the remaining athletes reported participating in additional sports (e.g., athletics, swimming, judo, fencing, archery, and skiing). BXC-only and multisport athletes did not differ significantly in lifestyle habits, except for leisure-time exercise, which was higher among multisport athletes (median 8.0 [IQR 5.75–10.0] vs. 5.0 [3.0–7.0] h/week; *p* = 0.021; r_rb = 0.38, 95% CI 0.08 to 0.66). The overall active lifestyle profile indicated regular engagement in physical activity beyond BXC, including leisure-time walking and exercise, whereas sitting time was higher during the workweek than on weekends (Table 2). Most participants reported never smoking (62.3%), 22.6% were former smokers, and current use of cigarettes or e-cigarettes was uncommon (Table 2). Furthermore, only about one-third of athletes (34.0%) reported current medication use (Table 2). Regarding sleep quality assessed via the B-PSQI, most of participants (60.4%) were classified as good sleepers, whereas approximately 40% met the criterion for poor sleep quality (Table 2).

In the exploratory subgroup comparison based on sport involvement, multisport athletes reported poorer sleep quality than BXC-only players, as indicated by higher B-PSQI scores (6.05 ± 3.12, median 6.0 [IQR 3.75–7.25] vs. 4.70 ± 3.73, median 4.0 [IQR 2.0–6.0], *p* = 0.05; r_rb = 0.32, 95% CI 0.02 to 0.60), and a higher prevalence of poor sleepers (55.0% vs. 30.3%).

Dietary habits of the study sample are summarized in Table 3.

In particular, fewer than half of the participants reported current use of dietary supplements, whereas most had not followed a specific diet during the previous year. Among those who had followed a specific diet, weight loss and ethical or religious reasons were the most frequently reported motivations (Table 3). Eating meals away from home was generally infrequent with 66.0% reporting that they did so rarely and 11.3% never, whereas most participants reported regular home meal preparation (Table 3). A varying frequency was detected in alcohol consumption, with wine and beer being the most commonly consumed beverages (Table 3). The AEBQ profile did not indicate marked dysregulation of eating behavior (Table 3). In particular, Enjoyment of Food showed the highest mean score, whereas Food Fussiness showed the lowest, with the remaining subscales displaying intermediate values (Table 3).

In the exploratory subgroup comparison based on sport involvement, multisport athletes showed significantly lower Satiety Responsiveness than BXC-only players (2.30 ± 0.79, median 2.25 [IQR 1.75–2.75] vs. 2.78 ± 0.71, median 2.75 [IQR 2.25–3.00], *p* = 0.02; r_rb = −0.38, 95% CI −0.66 to −0.07), whereas no other statistically significant differences in AEBQ dimensions were observed. When stratified according to the onset of visual impairment, athletes with acquired impairment showed higher scores in Emotional Overeating and Emotional Undereating than those with congenital impairment, although these differences did not reach statistical significance (2.62 ± 1.10, median 2.40 [IQR 2.00–3.00] vs. 2.16 ± 0.83, median 2.20 [IQR 1.40–2.60] and 3.29 ± 1.03, median 3.00 [IQR 2.80–4.00] vs. 2.48 ± 0.81, median 2.20 [IQR 2.00–3.00], respectively).

Table 4 presents the body appreciation data assessed by the BAS-2.

Body appreciation was generally high across the sample, as indicated by an overall average item mean score of 3.77 ± 0.12 and a total BAS-2 score of 37.68 ± 7.39. Particularly favorable mean scores were detected in the items regarding respect for one’s body and recognition of positive bodily qualities, followed by attention to bodily needs (3.85 ± 0.96), a positive attitude toward one’s body (3.83 ± 0.97), and feelings of love toward one’s body (3.81 ± 1.00). Conversely, the lowest scores were observed for appreciation of one’s unique physical characteristics (3.55 ± 1.16) and feeling beautiful despite differences from media images of attractive people (3.60 ± 1.23) (Table 4). No statistically significant differences in BAS-2 scores were observed between multisport and BXC-only athletes (3.84 ± 0.71, median 3.95 [IQR 3.43–4.33] vs. 3.72 ± 0.77, median 3.80 [IQR 3.20–4.00], *p* = 0.445; r_rb = 0.13) or between athletes with acquired and congenital visual impairment (3.78 ± 0.77, median 3.90 [IQR 3.20–4.18] vs. 3.76 ± 0.73, median 3.90 [IQR 3.50–4.30], *p* = 0.883; r_rb = 0.03).

## 4. Discussion

The present study provided an exploratory multidimensional characterization of health-related behaviors, such as eating behavior, body appreciation, and sleep quality, in a sample of competitive Italian BXC athletes. Overall, the findings describe a generally favorable lifestyle profile, although the cross-sectional design and use of external normative data do not permit a direct attribution of this profile to BXC participation. Of note, several of the observed outcomes were descriptively within ranges reported for healthy Italian adults, and in some cases, even slightly more favorable. This observation is particularly relevant considering that visually impaired individuals are generally exposed to a greater burden of physical inactivity, sedentary behavior [4,25], cardiometabolic risk [26], psychological distress, and social isolation compared to the general population [3,27,72,73]. Although the cross-sectional design does not allow causal inferences regarding the direct impact of BXC participation, the overall outcomes in this cohort are consistent with a potentially health-promoting role of regular participation in competitive adapted sports [74,75]. Participants reported physical activity beyond BXC, including walking and leisure-time exercise, indicating that BXC occurred within a broader active lifestyle. These data are noteworthy in a population for whom independent physical activity can be constrained by accessibility, transportation, environmental, and organizational barriers [7,8,76].

Regarding the anthropometric profile, most participants were classified as normal weight, while the proportion of individuals with obesity was relatively low and broadly compatible with that reported in healthy Italian adult reference populations [66]. However, BMI should be interpreted cautiously in athletic populations, as it does not distinguish between fat and lean mass and, in the present study, was calculated from self-reported height and weight. Despite barriers related to visual disability [49,50], most participants reported non-restrictive diets, frequent home meal preparation, and a low frequency of eating out. Nevertheless, these findings do not provide information about dietary quality, nutritional adequacy, or energy availability, which represent important parameters in athletes [42,77]. Such issues remain relevant in para-athletes, who may face additional challenges in translating general nutritional recommendations into individualized practices [48].

The AEBQ indicated no marked dysregulation of eating behavior, with high Enjoyment of Food and an overall profile that was descriptively comparable with reference values for healthy Italian adults [48]. Specifically addressing this potentially vulnerable population, such finding may be compatible with a beneficial association between regular sport participation and relatively balanced dietary habits [42]. However, because the AEBQ assesses behavioral tendencies rather than actual dietary intake, it cannot establish dietary quality or nutritional adequacy. Moreover, eating behavior is influenced by multiple personal, environmental, and behavioral factors, and nutritional knowledge alone does not necessarily translate into optimal dietary practices [44,45,46]. The significantly lower Satiety Responsiveness observed among multisport athletes compared to BXC-only athletes warrants careful interpretation and may reflect differences in sport participation, energy requirements, or training schedules rather than an adverse effect of multisport participation. Future studies should combine eating behavior measures with objective assessments of dietary intake and training load. Regarding the onset of visual disability, participants with acquired visual impairment showed numerically higher Emotional Overeating and Emotional Undereating scores than those with congenital visual impairment, although neither difference was statistically significant. These findings should, therefore, be regarded as exploratory and hypothesis-generating rather than evidence of a direct effect of visual impairment onset on eating behaviors. Interestingly, they suggest an area for further investigation regarding psychological adaptation to vision loss in larger cohorts [78].

Body appreciation represents another important dimension of the present findings. BAS-2 scores were high across all subgroups and aligned descriptively with healthy adult benchmarks [69]. Given the potential challenges associated with disability, altered body representation, and exposure to socially constructed appearance ideals, these data are noteworthy. In individuals with visual impairment, body representation may develop through multisensory experiences, proprioception, tactile information, and social interactions rather than visual information alone [40,55]. Of note, athletic identity and perceived physical functionality may further shape body perception in para-athletes [48]. Although high body appreciation may be compatible with positive psychosocial adaptation in this sample [40], the cross-sectional design prevents determining whether it is directly related to BXC participation or reflects pre-existing characteristics.

Sleep quality, which is fundamental to athletic recovery and closely associated with cognitive functioning, emotional regulation, immune function, and physical performance [47,56,57], represents another relevant outcome in the present study. Visual impairment, particularly the total loss of light perception, can disrupt circadian entrainment and predispose individuals to Non-24-Hour Sleep–Wake Rhythm Disorder [61]. Most of the athletes were classified as good sleepers, and the overall sleep profile and prevalence of poor sleepers fell within the normative ranges reported for the healthy Italian adult population [71]. While individual light perception status was not recorded in this survey, regular physical exercise and structured team routines may help stabilize sleep patterns [11,13,57,58]. The BXC-only group showed a mean B-PSQI score within the range reported for healthy Italian adults, while the multisport group exceeded the reference threshold for poor sleep [71]. This difference may reflect greater recovery demands associated with additional sport participation, as high training loads and insufficient recovery have been linked to poorer sleep in athletes [57,79,80]. Given that training-related variables were not specifically assessed in the present study, these findings do not provide direct evidence that multisport participation jeopardizes sleep quality. However, they suggest that sleep warrants particular attention when addressing competitive para-athletes [81].

Overall findings highlight adapted team sports as valuable and enjoyable settings for integrated health promotion interventions addressing nutrition, sleep, recovery, body appreciation, and physical performance. Sports organizations and multidisciplinary teams in this field should therefore implement digitally accessible, tailored nutritional counseling [48,49] and routine sleep hygiene screening, particularly for multisport para-athletes [57,79,81]. Social policies promoting adapted team sports to foster active living and psychosocial well-being among individuals with visual impairments are also needed.

Despite such encouraging findings, several limitations must be acknowledged. First, the cross-sectional design precludes causal inferences. Second, external normative data do not constitute a contemporaneous matched control group. Moreover, the relatively small subgroup sizes, together with the number of statistical comparisons performed across multiple outcomes, increase the possibility of chance findings. As no formal adjustment for multiple testing was applied, subgroup findings should be regarded as preliminary and exploratory and require confirmation in larger, adequately powered studies. The study also relied on self-reported measures and did not include objective assessments of dietary intake, energy availability, body composition, physical activity, training load, recovery, or sleep/circadian parameters. On this basis, future longitudinal studies should recruit larger samples and compare BXC players with visually impaired athletes participating in other adapted sports. Such a research focus will be essential to determine whether the favorable profile observed reflects a specific contribution of regular BXC practice, adherence to adapted sports in general, or, alternatively, pre-existing individual characteristics. Furthermore, our findings emphasize the critical need for greater direct involvement of athletes in scientific research. Actively engaging BXC players can foster a deeper appreciation of the vital role that empirical research plays in promoting, advocating, and driving the institutional growth of this adapted discipline. Direct participation is also essential to optimize response rates and ensure comprehensive data collection, capturing an accurate picture of the Italian BXC landscape across all regional teams and competitive levels.

## 5. Conclusions

In conclusion, competitive Italian BXC athletes—a growing yet understudied adapted team sport population—exhibit a favorable multidimensional lifestyle profile characterized by balanced eating behaviors, high body appreciation, and satisfactory sleep quality. These exploratory findings underscore the role of adapted team sports as inclusive settings for multidimensional health promotion among individuals with visual impairments.

## Figures and Tables

**Table 1:** Sociodemographic and anthropometric characteristics of the participants.

Variables	Visually Impaired Athletes (*n* = 53)
Age (years), mean ± SD (range)	39.3 ± 13.1 (18–67)
Sex, *n* (%)	
Male	41 (77.4)
Female	12 (22.6)
BMI Category, *n* (%)	
<18.5 (Underweight)	1 (1.9)
<25 (Normal weight)	33 (62.3)
25–29.9 (Overweight)	16 (30.2)
≥30 (Obese)	3 (5.7)
Classification of Visual Deficit, *n* (%)	
Visually impaired	8 (15.1)
Legally blind	45 (84.9)
Visual Impairment Onset, *n* (%)	
Congenital	33 (62.3)
Acquired	20 (37.7)
Educational Level, *n* (%)	
Primary school	1 (1.9)
Middle school diploma	11 (20.8)
High school diploma	28 (52.8)
University degree	13 (24.5)
Employment Status, *n* (%)	
Employed	28 (52.8)
Freelance	1 (1.9)
Student	9 (17.0)
Retired	7 (13.2)
Unemployed	7 (13.2)
Other	1 (1.9)

BMI, body mass index; SD, standard deviation.

**Table 2:** Lifestyle habits and sleep quality profiles of the study participants.

Variables	Visually Impaired Athletes (*n* = 53)
Current Sport Involvement, *n* (%)	
Single-sport (blind baseball)	33 (62.3)
Multisport	20 (37.7)
Blind baseball practice, hours/week, mean ± SD	3.49 ± 1.31
Household chores, hours/day, mean ± SD	2.15 ± 1.47
Flights of stairs climbed, flights/day, mean ± SD	5.16 ± 5.04
Sitting time (workweek), hours/week, mean ± SD	19.37 ± 18.42
Sitting time (weekend), hours/weekend, mean ± SD	9.75 ± 10.02
Leisure-time walking, hours/week, mean ± SD	12.92 ± 14.47
Leisure-time exercise, hours/week, mean ± SD	9.35 ± 12.14
Smoking Status, *n* (%)	
Never smoker	33 (62.3)
Former smoker	12 (22.6)
Current cigarette smoker	3 (5.7)
Current e-cigarette user	5 (9.4)
Current Medication Use, *n* (%)	
Yes	18 (34.0)
No	35 (66.0)
Sleep Quality Profile (B-PSQI), *n* (%)	
Good sleeper (B-PSQI ≤ 5)	32 (60.4)
Poor sleeper (B- PSQI > 5)	21 (39.6)

B-PSQI, Brief Pittsburgh Sleep Quality Index; SD, standard deviation.

**Table 3:** Dietary habits and Adult Eating Behavior Questionnaire scores of the study participants.

Variables	Visually Impaired Athletes (*n* = 53)
Current Dietary Supplement Use, *n* (%)	
Yes	23 (43.40)
No	30 (56.60)
Adherence to a Specific Diet (Past Year), *n* (%)	
Yes	14 (26.40)
No	39 (73.60)
Reason for Following a Specific Diet (*n* = 14), *n* (%)	
Medical reasons	1 (7.10)
Weight loss	7 (50.00)
Ethical or religious reasons	6 (42.90)
Frequency of Eating Meals Away from Home, *n* (%)	
Never	6 (11.32)
Rarely	35 (66.03)
Often	9 (16.95)
Almost always	3 (5.70)
Frequency of Home Meal Preparation/week, *n* (%)	
Never/almost never	7 (13.20)
Occasionally	8 (15.10)
About half of the time	3 (5.70)
Most of the time	12 (22.60)
Always/almost always	23 (43.40)
Frequency of Alcohol Consumption, *n* (%)	
Never	8 (15.10)
Once or less per month	5 (9.40)
2–4 times per month	21 (39.60)
2–3 times per week	14 (26.40)
4 or more times per week	5 (9.40)
Type of Alcoholic Beverages Consumed, (*n* = 45), *n* (%)	
Wine	28 (65.10)
Beer	29 (67.40)
Spirits	11 (25.60)
AEBQ Subscale Scores, mean ± SD	
Hunger	2.65 ± 0.65
Food responsiveness	2.47 ± 0.68
Emotional over-eating	2.33 ± 0.96
Enjoyment of food	3.71 ± 0.65
Satiety responsiveness	2.59 ± 0.77
Emotional under-eating	2.98 ± 1.03
Food fussiness	2.19 ± 0.81
Slowness in eating	2.88 ± 1.06

AEBQ, Adult Eating Behavior Questionnaire; SD, standard deviation.

**Table 4:** Mean scores for the Body Appreciation Scale-2 (BAS-2) items among visually impaired athletes (*n* = 53).

BAS-2 Items *	Mean ± SD
I respect my body	3.91 ± 0.79
I feel good about my body	3.79 ± 0.86
I feel that my body has at least some good qualities	3.91 ± 0.83
I take a positive attitude toward my body	3.83 ± 0.97
I am attentive to my body’s needs	3.85 ± 0.96
I feel love for my body	3.81 ± 1.00
I appreciate the different and unique characteristics of my body	3.55 ± 1.16
My behavior reveals my positive attitude toward my body	3.68 ± 0.82
I am comfortable in my body	3.75 ± 0.97
I feel like I am beautiful even if I am different from media images of attractive people	3.60 ± 1.23
Average item mean score	3.77 ± 0.12
Total score	37.68 ± 7.39

* Item responses were scored on a 5-point Likert scale ranging from 1 (“never”) to 5 (“always”); BAS-2, Body Appreciation Scale-2; SD, standard deviation.

## Data Availability

The datasets generated and/or analyzed during the current study are not publicly available due to privacy and confidentiality constraints; however, they can be obtained from the corresponding author upon reasonable request.

## Abbreviations

The following abbreviations are used in this manuscript:

BXC	Blind Baseball
AIBXC	Italian Blind Baseball Association
AEBQ	Adult Eating Behavior Questionnaire
BAS-2	Body Appreciation Scale-2
B-PSQI	Brief version of the Pittsburgh Sleep Quality Index
